# Kullback-Leibler Divergence-Based Differential Evolution Markov Chain Filter for Global Localization of Mobile Robots

**DOI:** 10.3390/s150923431

**Published:** 2015-09-16

**Authors:** Fernando Martín, Luis Moreno, Santiago Garrido, Dolores Blanco

**Affiliations:** Robotics Lab, Carlos III University, Madrid 28911, Spain; E-Mails: moreno@ing.uc3m.es (L.M.); sgarrido@ing.uc3m.es (S.G.); dblanco@ing.uc3m.es (D.B.)

**Keywords:** Markov chain Monte Carlo, Kullback-Leibler divergence, differential evolution, mobile robot, global localization, laser range finders

## Abstract

One of the most important skills desired for a mobile robot is the ability to obtain its own location even in challenging environments. The information provided by the sensing system is used here to solve the global localization problem. In our previous work, we designed different algorithms founded on evolutionary strategies in order to solve the aforementioned task. The latest developments are presented in this paper. The engine of the localization module is a combination of the Markov chain Monte Carlo sampling technique and the Differential Evolution method, which results in a particle filter based on the minimization of a fitness function. The robot’s pose is estimated from a set of possible locations weighted by a cost value. The measurements of the perceptive sensors are used together with the predicted ones in a known map to define a cost function to optimize. Although most localization methods rely on quadratic fitness functions, the sensed information is processed asymmetrically in this filter. The Kullback-Leibler divergence is the basis of a cost function that makes it possible to deal with different types of occlusions. The algorithm performance has been checked in a real map. The results are excellent in environments with dynamic and unmodeled obstacles, a fact that causes occlusions in the sensing area.

## 1. Introduction

The sensing system of a mobile robot plays a fundamental role in almost all tasks that it can perform. Some examples are: the navigation module should be able to detect obstacles; the object shape must be computed for manipulation; and the robot must estimate its own location to interact with the environment. The scope of this research is the global localization (GL) problem, which can be defined as the search of the robot’s coordinates in a known environment without information about the initial location. Two different types of systems are distinguished depending on the source of information: positioning systems and self-localization systems. The positioning systems rely on signals sent from external sources. The most common example is the Global Positioning System (GPS). In the self-localization systems, the information is provided by sensors implemented onboard the robot. Typical cases are localization modules for indoor environments where laser range finders compute two-dimensional (2D) or three-dimensional (3D) scans.

Regarding the available information about the robot’s pose (position and orientation), it is possible to distinguish between re-localization or tracking and GL. In re-localization, the initial pose is known (at least approximately). The pose is updated as accurately as possible while the robot is moving. The estimate based on proprioceptive sensors (odometry provided by the wheel encoders) is corrected by using local information from perceptive sensors (ultrasounds, laser scanners, vision, *etc*.). In GL, the initial location is unknown, and the search is not limited to a local area. The robot’s pose has to be estimated according to the global map, which is assumed to be known, and the local information provided by perceptive sensors.

Our recent work is focused on the development of a self-localization module for the experimental platform MANFRED-2 (http://roboticslab.uc3m.es/roboticslab/robot/manfred-2), fully developed by the Robotics Lab of the Carlos III University of Madrid. This platform is equipped with a laser range finder that gives the robot information about the environment. The laser scan provided by the sensing system is used to solve the GL problem.

Several GL modules based on the Differential Evolution (DE) method [1] have been designed in our previous work. DE is an optimization technique that relies on evolutionary computation concepts. Different versions have been proposed for 2D [2] or 3D [3]. The basic idea is that the robot’s pose is obtained from a population of possible estimates weighted by a fitness function that measures the difference between the true scan from the real location and the estimated scan from the population member or candidate solution. The population set is updated following the steps of the DE method in order to optimize the weights of the population members. The sensors’ information is integrated to obtain the solution of the GL problem, which is the population member with the best fitness value.

The Kullback-Leibler (KL) divergence [4] is applied in the most recent version of the filter [5] to implement a different cost function. While most fitness functions (in GL) are symmetric, the KL divergence is an asymmetric metric. Its asymmetry allows us to, by analyzing the sensing information, favor or penalize different situations. The parameters of the fitness function can be adjusted to improve the performance of the filter in some unexpected situations. In particular, the asymmetry is exploited to increase the robustness when there are occlusions caused by dynamic and/or unmodeled obstacles.

The Monte Carlo (MC) sampling method is an old concept devised by Ulam and Metropolis [6] that consists of representing a probability density function by a set of samples. It is a widely-used technique that admits multiple variations. The works of Metropolis [7] and Hastings [8] laid the foundation for a large class of sampling algorithms named Markov chain Monte Carlo (MCMC) [9]. In this type of method, the target distribution is approximated by a set of samples that explore the state space following a Markov chain mechanism. These samples are generated by successive jumps that depend on a transition probability.

Ter Braak has developed the DE-MC optimization filter [10], which is a method that combines MCMC and DE. He has applied his method to multiple optimization problems. In our recent research, we have designed a GL module based on the DE-MC algorithm [11]. Its main advantages are the improvement in the robustness (regarding the chances of success in the GL process) and the decrease of the population requirements with respect to the basic version (DE) of the filter.

In this work, a new version of the filter where the KL divergence-based cost function [5] is implemented together with the DE-MC GL algorithm [11] is presented. The main objective is to obtain a GL module that inherits the advantages of both approaches. This new version has been tested in a real map. As can be verified in the experimental results, the method performance is excellent in environments with occlusions, which is a very interesting characteristic that makes this technique a suitable approach for environments with dynamic objects and people. In addition, the population requirements are similar to those presented in [11].

The rest of this paper is organized as follows. First, the related work is reviewed in Section 2. After that, Section 3 details the KL divergence and the KL-based cost function. In Section 4, the main concepts about the algorithms used here (MC, MCMC, and DE) are explained. The GL filter is presented in Section 5. The experimental results are given in Section 6, and finally, the most important conclusions are outlined in Section 7.

## 2. Related Work

The GL problem can be solved following different approaches. In order to review the most significant techniques, a division is made between Bayesian-based, optimization-based and hybrid methods.

The Bayesian-based filters basically consist of two steps. The motion and perceptive information is incorporated into the *a posteriori* density function in the first step. After that, the robot’s pose is estimated in the second step following a specific criterion, such as the maximum density point or the average value. After convergence, the probability distribution is concentrated in a small area around the estimate. The most common methods included in this category are the particle filters. Grid-based probabilistic filters [12] and MC localization methods [13] are some interesting examples that follow these ideas. A Markov localization module for dynamic environments has been implemented by Fox *et al.* [14]. The variation of MC developed by Thrun *et al.* [13] is applied to GL and re-localization.

The refinement of the hypotheses has been studied by several authors to improve this group of techniques. The aim is to decrease the number of particles needed by the localization filter. The corrective gradient refinement method was introduced by Biswas *et al.* [15]. They reduce the population requirements by using gradients of the observation model. Another option that includes the movement of the robot and the most recent observation in the proposal distribution was developed in [16]. Zhang *et al.* [17] have created the self-adaptive MC localization (SAMCL) algorithm. They distribute more efficiently the samples after calculating what they have called the similar energy region.

The key of the optimization-based algorithms is the fitness function that is minimized in each motion-perception cycle. This fitness function has to include the available information (motion and perception). These methods are usually population-based, and each member of the population set is a possible solution to the GL problem. The estimate will be the element of the population with the best fitness value. The localization task is basically considered as an optimization problem that can be solved following different strategies. For example, the Kalman filters use the derivative of the cost function to estimate the robot’s pose. Their computational cost is low, but they cannot manage multi-hypotheses problems. Kalman filters are optimum solutions for tracking once the robot is correctly localized [18]. Another idea is to execute a stochastic search to find the best solution. There are multiple families of algorithms that rely on this assumption: DE, genetic algorithms (GA), particle swarm optimization (PSO), ant colony optimization (ACO), *etc*. A review of these methods is presented in [19,20]. Lisowski [21] has combined DE and MC to develop a localization module. The harmony search algorithm [22] has been used by Mirkhania *et al.* [23] to design a GL module. A variant of the MC algorithm that includes a genetic algorithm optimizer has been proposed by Luo *et al.* [24]. The core of the GL method presented in this paper is a combination between the MCMC sampling approach and the DE method. The specific state of the art of these techniques is reviewed in Section 4.

The hybrid methods, or multi-hypotheses Kalman filters [25,26], are a different group of techniques where the set of solutions is formed by normal (Gaussian) probability distributions. In this case, the creation or elimination of solutions is not purely Bayesian. Each probability distribution is guided by a Kalman filter. They usually rely on a decision tree search mechanism based on geometric constraints together with probabilistic attributes to manage the global data association problem. For example, laser scans are used to estimate the robot’s pose in [27]. They use a set of Gaussians to model the likelihood function according to the available information. Kalman and particle filters are combined in [28].

The quadratic error between the real observation vector and the estimated scan from the candidate is frequently used to compute the fitness value. Other options have been suggested by different researchers. The Manhattan distance is applied in our previous work to improve the method performance when there are dynamic obstacles [29]. The Hausdorff distance has been considered by Donoso *et al.* [30]. The “entropy of future belief distributions” is exploited by Fox *et al.* [31]. A feature-based technique that relies on the Mahalanobis distance has been developed by Arras *et al.* [32]. In [5], it was concluded that the KL divergence is an appropriate metric for environments with occlusions and/or unmodeled obstacles.

## 3. Kullback-Leibler Divergence

The optimization method that is applied here to develop the GL module is based on the minimization of a fitness value. The KL divergence is the tool that is used to implement the fitness function. This metric and the fitness function are derived in this section.

The definition of the KL divergence was given by Kullback and Leibler in 1951 [4]. It is a “a non-symmetric measure of the difference between two probability distributions *P* and *Q*”. It can be defined in discrete spaces using the following formula:

(1) $$d_{KL}\left(P||Q\right)=\sum_{i}p\left(i\right)\ln\frac{p(i)}{q(i)}$$

where *p* and *q* are the probability densities of *P* and *Q*. This definition is valid when the densities add up to one ($\sum_{i}p\left(i\right)=\sum_{i}q\left(i\right)=1$). Each pair of densities are included in the formula only if q(i)>0 and p(i)>0. *P* is often referred to the ‘true’ probability distribution (for example, the real observation vector), and *Q* represents an approximation of *P* (for example, the expected measurements from the candidate solution).

The KL divergence from *P* to *Q* is not equal to the KL divergence from *Q* to *P*. It represents the average of the logarithmic difference between *P* and *Q*, where the average is weighted by *P*.

### 3.1. Kullback-Leibler Divergence between Two Scans

In this work, the 2D map of the environment is divided into a grid of regular-sized cells. Each cell has an associated value in the interval [0,1] that represents the probability of being occupied. The following notation is used to represent the map:

(2) $$m=\{m_{ij}:\;1\leq i\leq n,\;1\leq j\leq o\}$$

where each cell is defined by $m_{ij}$, *n* and *o* are the map dimensions and *i* and *j* are positive integers. The probability of being occupied is $p(m_{ij})$. It is assumed that the probabilities are known.

The sensor measurements register the information that the robot receives about the environment. In the sensing system used here, the distances to the closest obstacles and the cells crossed by the laser beams are computed.

Two laser scans are compared to calculate the fitness value of each candidate solution. The first one is formed by the real laser readings from the robot’s true location, and the second one is estimated from the candidate solution (if the map is known, it is possible to estimate the readings from specific poses).

Before introducing the formula of the KL divergence for this particular problem, several variables have to be defined. The robot’s true location is $x={\left(x,y,\text{θ}\right)}^{T}$, and the laser scan from x is $z=(z_{1},z_{2},...,z_{N_{s}})$. The estimate from the candidate solution $\hat{x}$ is $\hat{z}$ (measurements obtained using *m*). The area (in cells) covered by an observation z from pose x is represented by $S\left(x,z\right)=\left\{m_{ij}^{x,z}\right\}$. Using this notation, the KL divergence for the laser beam *k* can be defined as:

(3) $$d_{KL}^{k}(P_{S(x,z_{k})}\left|\right|P_{\hat{S}\left(\hat{x},{\hat{z}}_{k}\right)})=\sum_{i,j\in S_{T}}p_{S(x,z_{k})}\left(m_{ij}\right)\ln\frac{p_{S(x,z_{k})}\left(m_{ij}\right)}{p_{\hat{S}\left(\hat{x},{\hat{z}}_{k}\right)}\left(m_{ij}\right)}$$

where $S_{T}$ is the maximum between $S(x,z_{k})$ and $\hat{S}\left(\hat{x},{\hat{z}}_{k}\right)$. It can be noticed that the numbers of cells crossed by $S(x,z_{k})$ and $\hat{S}\left(\hat{x},{\hat{z}}_{k}\right)$ are different. It has been assumed that these areas end at the first unknown cell.

To simplify the notation, the following expression will be used from now on:

(4) $$d_{KL}^{k}(P_{S_{k}}\left|\right|P_{{\hat{S}}_{k}})=\sum_{i,j\in S_{T}}p_{S_{k}}\left(m_{ij}\right)\ln\frac{p_{S_{k}}\left(m_{ij}\right)}{p_{{\hat{S}}_{k}}\left(m_{ij}\right)}$$

**Figure 1:**
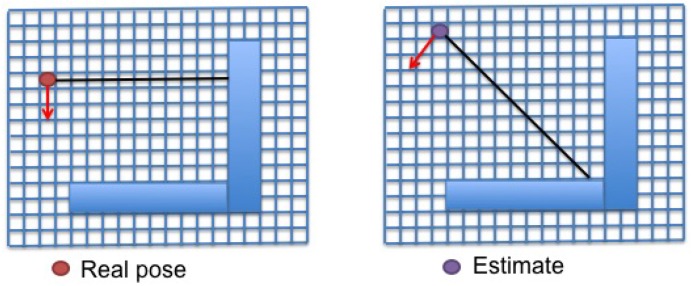
Single measurements obtained by a laser scanner. (**Left**) Real pose; (**Right**) estimated pose. The line representing the laser beam.

Equation (4) is applied to compare real observations and estimates with the same bearing to compute the KL divergence for a given orientation. An example is shown in Figure 1 and Table 1. Single measurements from the true pose (left) and the candidate (right) are displayed in the figure. The probability distribution of the laser beam has to be defined. Different options are proposed in this work (Section 3.2 and Section 3.3). A simple model is presented in the table to illustrate this example. Each cell crossed by the laser beam is numbered in increasing order. The map probabilities are divided into free space (0.05), obstacles (0.95) and unknown space (0.5). Thirteen single values are computed for the probability distributions from the real pose ($p_{1}$) and the estimated one ($p_{2}$), because the longest laser beam crosses 13 cells. The KL divergence, which is the sum of the terms of the third row, is 0.3445.

**Table 1:** KL computation for the beam orientation shown in Figure 1. Types of cells: free space =0.05, obstacle =0.95, unknown space =0.5. $p_{1}\left(cell\right)=p_{S_{k}}\left(m_{ij}\right)$, $p_{2}\left(cell\right)=p_{{\hat{S}}_{k}}\left(m_{ij}\right)$.

Cell	1	2	9	10	11	12	13
$p_{1}\left(cell\right)$	0.05	0.05	0.05	0.05	0.05	0.95	0.5
$p_{2}\left(cell\right)$	0.05	0.05	0.05	0.95	0.5	0.5	0.5
$p_{1}\left(cell\right)\ln\frac{p_{1}\left(cell\right)}{p_{2}\left(cell\right)}$	0	0	0	−0.1472	−0.1151	0.6098	0

If the laser scan is formed by $N_{s}$ observations, the KL divergence for the whole scan will be:

(5) $$d_{KL}(P_{S}\left|\right|P_{\hat{S}})=\sum_{k=1}^{N_{s}}\sum_{i,j\in S_{T}}p_{S_{k}}\left(m_{ij}\right)\ln\frac{p_{S_{k}}\left(m_{ij}\right)}{p_{{\hat{S}}_{k}}\left(m_{ij}\right)}$$

Since each laser reading is independent from the others, the KL divergence is the sum of the divergences of the individual measurements.

Analyzing Equation (5), $d_{KL}$ is greater or equal to zero. It will be equal to zero when the laser scan from the real pose is equal to the estimated scan from the candidate pose. The GL module will evolve to minimize $d_{KL}$, which will be the basis of the fitness value associated with each member of the population.

When using a symmetric metric, such as the L2-norm, the cost value is given by the sum of the squared errors (between true measurements and estimates). In this case, there is no flexibility to modify the fitness function. When considering the KL divergence, each measurement is defined by a probability distribution, and it is possible to model the distribution to improve the method in some situations.

### 3.2. Sensor Probability Models

The KL divergence is dependent on the profile of the probability distributions. In this section, these profiles are selected according to the sensing system for the particular problem addressed here.

A one-dimensional probability distribution that depends on the range measurement in the beam direction must be defined for a single beam of the laser scan ($z_{k}$). The occupancy probability of those cells crossed by the laser beam can be updated when the sensor information is received.

In [33], the authors propose to use Bayesian models to approximate range finders in dynamic environments. They obtain p(z|x,m), which is the probability of reading z in the map *m* when the robot’s pose is x. In an ideal case without noise, a theoretical function *h* could be chosen to represent the observations (z=h(x,m)). However, different sources of noise can be identified in this type of system: measurement noise of the laser device, inaccuracies in the observation model and inaccuracies in the robot’s pose estimate. These aspects have to be included in the probabilistic model.

Other options to model sensor measurements are discrete grid maps [14,34] or continuous metric maps [35]. Among them, the technique proposed by Moravec [34] relies on non-Gaussian densities over a discrete grid of possible distances measured by a sonar. In [14], Fox *et al.* have taken into account only the distance to the closest obstacle in the sensing direction. They consider that the measurements can correspond to modeled or unmodeled obstacles. Two more causes are added by Thrun *et al.* [35]: people moving around the robot and a maximum range measurement originated when the obstacles are beyond the sensor range.

Three different events are taken into account in the probabilistic model of the sensors. A probability function $p_{hit}$ is originated by the modeled obstacles (measurement of the laser scanner). The unmodeled obstacles have to be considered, thus a different function $p_{occ}$ is added to represent possible occlusions. It is necessary to model the distances behind the obstacles because the cost function compares pairs of measurements, and one of them will be longer than the other one. Since there is no information about this area of the map, it must remain unknown. $p_{unkn}$ is chosen to define these unknown zones. All of this information is mixed together to generate the probability density function for a laser beam:

(6) $$p\left(z_{k}|x,m\right)=k_{h}p_{hit}\left(z_{k}|x,m\right)+k_{o}p_{occl}\left(z_{k}|x,m\right)+k_{u}p_{unkn}\left(z_{k}|x,m\right)$$

The individual probabilities can vary between zero and one. $k_{h}$, $k_{o}$ and $k_{u}$ are the weights of each distribution. By choosing different values depending on the relation between the real distance and the estimate, it will be possible to penalize or favor the fitness value in some situations. This fact, which will be explained in Section 3.3, will allow the GL method to obtain a better performance in an environment with occlusions or dynamic obstacles.

The probability densities are defined in the following way. A Gaussian distribution is used to represent the laser reading. If the real distance measured by the sensor is $z_{t,k}^{*}$, this distribution is given by:

(7) $$\begin{array}{ccc} p_{hit}\left(z_{k}|x,m\right) & = & \frac{1}{\sqrt{2\pi \sigma_{hit}^{2}}}e^{-\frac{{\left(z_{k}-z_{k}^{*}\right)}^{2}}{2\sigma_{hit}^{2}}} \end{array}$$

where $\sigma_{hit}^{2}$ is the noise variance. A Gaussian is usually chosen to represent the laser range finders’ noise.

A uniform distribution is chosen for the unmodeled obstacles. This probability represents unexpected obstacles that generate shorter readings with respect to those expected according to the map. For example, it happens when there are people moving around the robot. The unknown zones are also represented by a uniform distribution from the measurement up to the maximum observation range.

**Figure 2:**
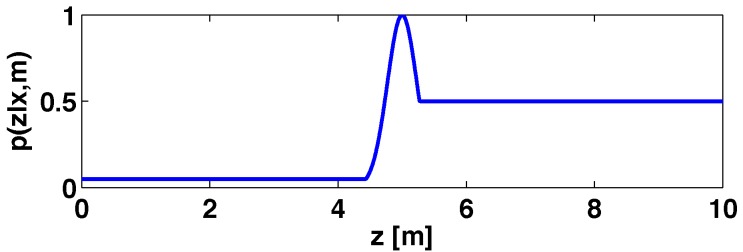
Probability distribution for a laser beam.

An example of a probability profile is shown in Figure 2. The reading provided by the laser device is 5 m, thus $p_{hit}$ is a Gaussian distribution with five themean ($k_{h}$ being one). Possible occlusions (unmodeled obstacles) are represented by a uniform distribution with a low value ($k_{o}=0.05$) when the distance is smaller than the laser measurement. The unknown places, located between five and the maximum distance that can be measured by the sensors, are modeled with $k_{u}=0.5$.

Equation (6) is applied to the real readings. The fitness value is obtained by comparing real and estimated observations. There is an equivalent formula for the probability distribution of the estimated measurements:

(8) $$p\left({\hat{z}}_{k}|\hat{x},m\right)={\hat{k}}_{h}p_{hit}\left({\hat{z}}_{k}|\hat{x},m\right)+{\hat{k}}_{o}p_{occl}\left({\hat{z}}_{k}|\hat{x},m\right)+{\hat{k}}_{u}p_{unkn}\left({\hat{z}}_{k}|\hat{x},m\right)$$

To be coherent with the KL definition, Equation (6) and Equation (8) are normalized to add up to one. The normalization factor is not included for simplicity. These equations are the basis of the GL module cost function.

### 3.3. KL-Based Fitness Function

**Figure 3:**
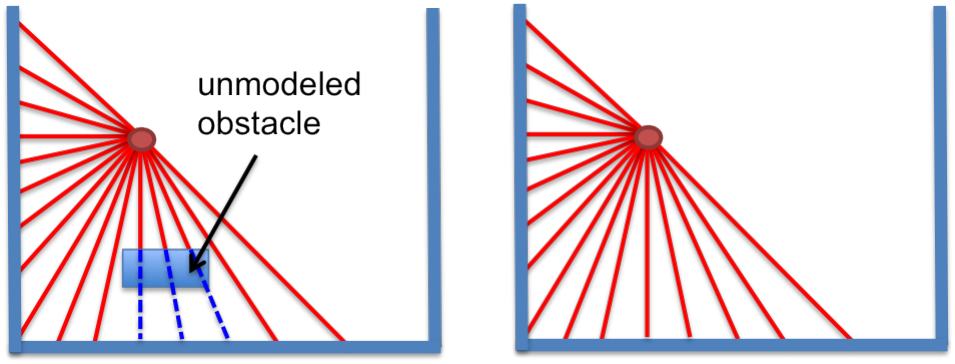
(**Left**) Unmodeled obstacle that generates an occlusion; (**Right**) laser estimate. Laser beams readin the known map and those not contained in the real observation vector in dashed lines.

Unmodeled obstacles generate an error between the real distance read by the laser range sensor and the estimated distance that is based on the known map. An example is presented in Figure 3. The real measurements (left) perceive the obstacle that is not modeled in the map, but the laser readings from the optimum estimate in the map (right) do not see the obstacle. In these conditions, an occlusion error will appear, and the localization process will be harder.

The coefficients of Equation (6) and Equation (8) are defined depending on different options to manage possible occlusions or impossible situations. If the real reading is lower than the estimated one, the wrong locationcan be due to an occlusion that has to be taken into account. When the estimate is lower than the true reading, it is an impossible situation that cannot occur when the estimate is the optimum one. The formula of the cost function has to be penalized in these situations to discard wrong locations.

The cost value of the GL filter is computed according to Equation (5). It uses the KL divergence to compare two probability distributions ($P_{S}$ and $P_{\hat{S}}$) for each reading (*k*) of the laser scan. The probability profiles follow Equation (6) and Equation (8). The coefficients of these equations are detailed in Table 2.

**Table 2:** Values for the coefficients of Equation (6) and Equation (8) depending on the relation between the real measurement ($z_{k}$) and the estimated one (${\hat{z}}_{k}$).

	$z_{k}<<{\hat{z}}_{k}$	$z_{k}\leq {\hat{z}}_{k}$	$z_{k}>{\hat{z}}_{k}$	$z_{k}>>{\hat{z}}_{k}$
$k_{o}$	0.1	0.1	0.1	0.95
${\hat{k}}_{o}$	0.05	0.05	0.05	0.05
$k_{h}$	0.9	0.9	0.9	0.95
${\hat{k}}_{h}$	0.95	0.95	0.95	0.05
$k_{u}$	0.15	0.5	0.9	0.95
${\hat{k}}_{u}$	0.5	0.5	0.5	0.05

If there is an occlusion, the real measurement will be much lower than the estimate (Figure 3). This situation is registered in the left column of the table. The objective is to favor the cost. In this case, $k_{u}$ is fixed to 0.15. The low value selected for this parameter increases the robustness to occlusions, because it makes the cost value not be penalized.

If the real laser reading is slightly lower or similar to the estimated one ($z_{k}\leq {\hat{z}}_{k}$), the difference between distances can be caused by an occlusion or by the laser measurement noise. In this case, the fitness function is penalized a little bit ($k_{u}=0.5$). It can be noticed that the influence of the coefficients is null when $z_{k}={\hat{z}}_{k}$.

If the laser reading is $z_{k}>>{\hat{z}}_{k}$ when the estimate is the optimum one, the measurement is probably wrong (see Figure 3 to analyze this option), because it is an impossible situation (from the correct location), and the fitness function has to be strongly penalized. $d_{KL}$ has to present a high value to discard this candidate; thus, $k_{o}$, $k_{h}$ and $k_{u}$ are set to a high value (0.95), and ${\hat{k}}_{h}$, ${\hat{k}}_{o}$ and ${\hat{k}}_{u}$ are fixed to a low one (0.05). If the difference is smaller ($z_{k}>{\hat{z}}_{k}$), the solution is not correct, but it can be close to the optimum one. In this case, only the unknown cells are penalized ($k_{u}=0.9$). The reader can notice that the coefficients have been empirically chosen to deal with occlusions. Different values will cause different behaviors.

The last element included in the cost function is a correction factor to distinguish between places with and without occlusions when the same fitness value is obtained:

(9) $$KLD=d_{KL}(P_{S}\left|\right|P_{\hat{S}})e^{\frac{N_{occ}}{N_{s}}}$$

where $N_{occ}$ is the number of occlusions ($z_{k}<<{\hat{z}}_{k}$). KLD is the cost value that is minimized by the GL filter to obtain the robot’s location.

## 4. Markov Chain Monte Carlo and Differential Evolution

The GL module is based on two different methods that will be explained in this section. First, an overview of the MC sampling technique is given. After that, MCMC and DE are introduced. Different references are included for a more detailed explanation.

As a reminder, the goal is to estimate the robot’s pose using the available information (2D laser scan). A population set where each member is a candidate solution will be defined. A simulated observation vector can be computed from each candidate solution because the map is known. The observations from the candidate will be compared to the real laser readings from the true location to compute the cost value. Since the fitness value can be associated with a probability, the purpose of the GL filter is to find the solution maximizing the probabilities of the population.

### 4.1. Monte Carlo Sampling

In robotics, the localization problem can be solved with particle filters. MC is a sampling technique that is very often applied in particle filters. It consists of drawing an “i.d.d. set of samples ${\left\{x_{i}\right\}}_{i=1}^{N_{P}}$ from a target density p(x) defined on a high-dimensional space X” [36] (e.g., the space of possible locations). The probability distribution is approximated by the population set:

(10) $$p_{N_{P}}\left(x\right)=\frac{1}{N_{P}}\sum_{i=1}^{N_{P}}\delta_{x_{i}}\left(x\right)$$

where $\delta_{x_{i}}\left(x\right)$ represents the Dirac delta mass associated with the candidate $x_{i}$ and $N_{P}$ is the population size. The probability mass of each member is $1/N_{P}$.

Equation (10) represents the most basic version of the MC method. It will only succeed if the probability distribution is very simple (for example, Gaussian). In [36], Andrieu *et al.* mention different variations that can be applied to more complicated distributions: rejection sampling (RS), importance sampling (IS) and sampling importance resampling (SIR).

The main idea of the IS strategy [37,38] is to include weights *w* to set the importance of each particle:

(11) $$p_{N_{P}}\left(x\right)=\sum_{i=1}^{N}w\left(x_{i}\right)\delta_{x_{i}}\left(x\right)$$

This sampling method still presents shortcomings. First, weights in particles with low/high probabilities are drastically reduced/increased, which can cause a fast degeneration of the particles. A variant proposed to deal with this issue is the SIR strategy [39]. In the SIR method, particles with higher weights are replaced by a set of particles of equal weights around the original position of the higher weight particles, and particles with lower weights are removed. The same weight is given to all particles in the resampling stage. Second, an area is not evaluated if there are no particles. The number of particles must be increased to explore the whole map. Larger sets imply worse computational costs. In particular, the population requirements can be huge in GL. Third, new sensor information has to be included in each iteration to modify the weights, thus many motion-perception cycles would be needed to solve the GL problem.

Studying these characteristics, MC will be very efficient in re-localization, but it can be inefficient in GL, because a high number of particles is needed to make an adequate approximation of the density function. In addition, a high number of motion-perception cycles is required until convergence. The most significant advantages are the statistical robustness and the performance depending on the noise level.

Obtaining distributions that are easy to sample from and good approximations of the state space at the same time is almost impossible in some problems [36]. If the topic addressed here is analyzed, the available information is the laser scan perceived by sensors and the map of the environment. The state space is formed by all possible poses. The probability distribution associated with the GL problem is very complicated to sample from, because it depends on the sensor measurements and the geometry of the environment. Other sampling methods based on Markov chains can be used in these cases.

Besides, the jumping step of the DE-MC technique will help the localization process regarding the population size that is needed to cover the whole space at the initial stages (see Section 5).

### 4.2. Markov Chain Monte Carlo-Metropolis-Hastings Method

The MCMC algorithms have surged as an interesting variation of the RS idea [9]. They explore the state space using a Markov chain mechanism. The Markov chain consists of a set of $N_{P}$ particles that are generated by successive jumps to approximate a target distribution p(x).

The most popular MCMC method is the Metropolis–Hastings algorithm (MH) [8]. Its basic version is based on the comparison of a random value $u∼U_{\left(0,1\right)}$ and a trial sample $x_{i*}∼q\left(x_{i*}|x_{i}\right)$, where q(x) is another distribution (easier to sample than *p*) that is used to sample p(x) (for instance, a Gaussian). The following accept/reject approach is applied to accept the trial sample:

(12) $$u<A\left(x_{i},x_{i*}\right)=min\left\{1,\frac{p\left(x_{i*}\right)q\left(x_{i}|x_{i*}\right)}{p\left(x_{i}\right)q\left(x_{i*}|x_{i}\right)}\right\}$$

This acceptance probability selects the next element of the chain. If the trial is accepted, the next element is $x_{i+1}=x_{i*}$; otherwise, the candidate is rejected and $x_{i+1}=x_{i}$.

An example of an application is given to illustrate these concepts. The Markov chain can be defined in different ways. For example, each sample can be a possible solution of the GL problem (robot’s pose). After generating a trial sample, the probability that is used to compare both solutions will depend on the cost value. If the probabilities associated with the current sample and the trial are $\pi (x_{i})$ and $\pi (x_{i*})$, respectively, the acceptance probability could be:

(13) $$u<A\left(x_{i},x_{i*}\right)=min\left\{1,\frac{\pi (x_{i*})}{\pi (x_{i})}\right\}$$

The Markov chain characterized by Equation (13) will evolve to the poses that maximize the cost function. However, this operator will be defined here in a different way, because the objective is to minimize the fitness function. The acceptance operator of this GL module will be detailed in Section 5.

In the MH method, it is critical to choose an adequate proposal distribution $q(x_{i*}|x_{i})$. Two conditions must be satisfied to ensure convergence: aperiodicity (no cycles) and irreducibility (positive probability of reaching all states in a finite number of steps). The main drawbacks of this method are the local-trap problem in systems whose landscape has multiple basins and the difficulty to sample from distributions with difficult or even intractable integrals.

More recent variants of the MCMC method called population-based MCMC are not sequential, but parallel. Different Markov chains that can follow different distributions are run in parallel to solve the optimization problem. The local-trap problem is reduced in these versions. These methods also allow one to share information between chains, learning from past samples and improving the convergence speed. Some examples are: adaptive direction sampling [40], conjugate gradient Monte Carlo [41], parallel tempering [42], evolutionary Monte Carlo [43] and equi-energy sampler [44].

Many authors have combined evolutionary algorithms and population-based MCMC methods: Ter Braak [10], Linage and Wong [43], Liang [45], Laskey and Myers [46], *etc*. The method proposed by Ter Braak, which combines MCMC and DE, is applied to many optimization problems, concluding that the simplicity, speed of calculation and convergence are improved when compared to the original MCMC technique. In our previous work [11], the MH version of the population-based MCMC algorithm was combined with the DE evolutionary technique according to the method proposed by Ter Braak to design a GL module.

### 4.3. Differential Evolution Algorithm for GL

The DE method [1] has been applied in our previous work to develop several GL modules [2,3]. This technique is summarized in this section.

The initial population is formed by $N_{P}$ candidates that are generated randomly to cover the whole map. Each candidate $x_{i}^{k}$ is a possible solution to the GL problem (robot’s pose at iteration *k*). The other input parameters are the laser scan from the true location, the known map and the DE internal parameters. An initialization method to determine an adequate population size can be found in [47].

The candidates are evaluated by a fitness function that compares an estimated scan from the candidate pose to the real measurements from the true location. The objective is to estimate the robot’s pose that minimizes the fitness value. In this document, the fitness value is calculated by Equation (9).

The main algorithm is executed until convergence. The evolutionary search contains three stages: mutation, crossover and selection. In a single iteration, a new population is created for the next generation, evolving to the robot’s pose that minimizes the fitness value.

Each population member is mutated according to the following equation:

(14) $$x_{i*}^{k}=x_{r_{0}}^{k}+F\left(x_{r_{1}}^{k}-x_{r_{2}}^{k}\right)$$

where $x_{i*}^{k}$ is the mutated vector and $x_{r_{0}}^{k}$, $x_{r_{1}}^{k}$ and $x_{r_{2}}^{k}$ are parameter vectors chosen randomly from the population at iteration *k* and are different from the running index. The scale factor F∈(0,1) is a real and constant coefficient that controls the amplification of the differential variations $\left(x_{r_{1}}^{k}-x_{r_{2}}^{k}\right)$.

The crossover operator increases the diversity of the next generation. Each parameter of the crossed vector $s_{i}^{k}={\left(s_{i,1}^{k},s_{i,2}^{k},\ldots ,s_{i,D}^{k}\right)}^{T}$ is selected from a component of the mutated vector ($x_{i*,j}^{k}$) or the current population ($x_{i,j}^{k}$) according to the next rule:

(15) $$s_{i,j}^{k}=\left\{\begin{array}{cc} x_{i*,j}^{k} & \mathrm{if}\;p_{i,j}^{k}<\delta \\ x_{i,j}^{k} & \mathrm{otherwise} \end{array}\right.$$

where $p_{i,j}^{k}$ is a random value in the interval [0,1] that is generated for each parameter *j* of the population member *i* at step *k*, and δ is the crossover probability that controls the crossover rate. The number of chromosomes *D* is equivalent to the dimensions of the state space, which is three in this problem.

The selection stage compares the mutated and crossed vector $s_{i}^{k}$ to the current population member $x_{i}^{k}$ to choose the best candidate for the next generation. If the vector $s_{i}^{k}$ yields a better value for the fitness function than $x_{i}^{k}$, then it is replaced by $s_{i}^{k+1}$; otherwise, the old value $x_{i}^{k}$ is retained for the new generation.

When the method converges after a number of iterations, it returns the solution of the GL problem, which is the final population member with the lowest fitness value.

## 5. KL-Based Differential Evolution Markov Chain GL Filter

The core of the KL-based DE-MC GL filter is explained in this section. It relies on the same concepts applied by Ter Braak [10] to transform the $N_{P}$ particles of the DE method into $N_{P}$ Markov chains. The pseudocode is presented in Algorithm 1.

**Algorithm 1** KL-based DE-MC GL module.
1:**function** *DE_MC_GL*(**z**, *m*, *conf_parameters*)2:     **for** *i* = 1 : *N_P_* **do**                            ▷ Initialization of *N_P_* Markov chains3:        $x_{i}^{0}=uniform\left(free\_map\right)$4:**     end for**
5:     j=16:     **while** (CONVERGENCE CONDITIONS) **do**7:        **for** $i=1:N_{P}$ **do**8:             $x_{i*}^{j}=x_{i}^{j}+F\left(x_{r_{1}}^{j}-x_{r_{2}}^{j}\right)+e$                                         ▷ Mutation9:             $r_{log}=fitness\left(x_{i*}^{j}\right)-fitness\left(x_{i}^{j}\right)$10:             $u∼U_{\left(0,1\right)}$11:             **if** $r_{log}<\log u$ **then**                     ▷ Selection, next sample of each chain12:                $x_{i}^{j+1}=x_{i*}^{j}$13:             **else**14:                $x_{i}^{j+1}=x_{i}^{j}$15:             **end if**16:        **end for**17:        j←j+1                                                ▷ Next iteration index18:        $optimum\_location=x_{i}^{j}:\min\left\{fitness\left(x^{j}\right)\right\}$19:        conv_conditions_checking(...)20:     **end while**21:**end function**                                                      ▷ Return solution


At the beginning, the initial population is spread uniformly to cover the free places of the known map (Lines 2–4 of Algorithm 1). Each candidate represents a Markov chain where new potential samples are created in each iteration.

In the DE-MC optimizer, the new potential samples are generated by the mutation stage of the DE method. After that, the new candidates are accepted or rejected following a selection mechanism. An advantage of this approach is that it exploits the exploratory efficiency of the DE method to run the exploration jumps and the statistical efficiency of the MC RS strategy via the Metropolis ratio, which defines the acceptance probability. In other words, the DE method is applied to the jumping step of the MCMC sampling technique. This modification solves an important aspect of MCMC in real parameter spaces, which is the choice of a suitable scale and orientation for the jumping distribution. This problem is only solved in orientation, but not in scale when applying other adaptive direction sampling techniques.

Analyzing the original DE method (Section 4.3), the mutation step combines three random vectors, and after that, the crossover operator is used to generate the new proposals. The fitness value of the new candidate ($s_{i}^{k}$) is compared to the fitness value of the current vector ($x_{i}^{k}$) to select the members of the next generation. In other words, the new candidate is accepted if $r=\pi \left(s_{i}^{k}\right)/\pi \left(x_{i}^{k}\right)>1$ (note that π refers to probabilities and fitness is used for the fitness values). The evolution of the population set optimizes the fitness function.

Different researchers have concluded the generation of new samples, and their acceptance criterion must satisfy the “balance condition” to make an adequate conversion from the DE mechanism to a population-based MCMC sampling [48,49,50]. This requirement means that if a sample $x_{i}^{j}$ is drawn from the target distribution, the next sample $x_{i}^{j+1}$ must be drawn from the same target distribution, possibly dependent on $x_{i}^{j}$. This condition cannot be satisfied with Equation (14).

Ter Braak proposes the next option to generate the new samples:

(16) $$x_{i*}^{j}=x_{i}^{j}+F\left(x_{r_{1}}^{j}-x_{r_{2}}^{j}\right)+e,\;\;\;\;\;\;e∼N{\left(0,b\right)}^{d}$$

where e is a symmetric normal distribution in a *d*-dimensional space that is added to guarantee that the whole parameter space is covered. *b* is small when compared to the variance of the target. *F* is set to 0.7 in the experiments according to the study made in [29]. Note that *j* is used instead of *k* to distinguish between the iterations of the new method (Markov chains) and the iterations of the old version. Equation (16) is applied in this GL filter to create $N_{P}$ new candidates (Line 8).

The crossover operator is not included because the objective is to keep the method close to the basic concept of a population-based MCMC algorithm.

The acceptance criterion adopted by Ter Braak is based on a probabilistic rule. $x_{i*}^{j}$ is accepted with probability min(1,r), where $r=\pi \left(x_{i*}^{j}\right)/\pi \left(x_{i}^{j}\right)$. This mechanism has to be modified due to the properties of the fitness function. In this work, the sensor properties are exploited according to the concepts explained in Section 3 to compute the the fitness value given by Equation (9). Therefore, it is not possible to measure direct probabilities. Whereas the objective of the original DE-MC technique is to maximize the probability, the fitness value has to be minimized in this filter.

The acceptance operator (Lines 9–15) follows an idea shown in an example in [10]. It relies on the difference between the fitness value of the proposal and the current member:

(17) $$r_{log}=fitness\left(x_{i*}^{j}\right)-fitness\left(x_{i}^{j}\right)$$

This difference is compared to the logarithm of the random number $u∼U_{\left(0,1\right)}$ to define an empirical acceptance criterion. The new member is accepted if $r_{log}<\log u$; otherwise, it is rejected. It can be noticed that logu is in the interval (−∞,0) and $r_{log}$ is negative when the fitness value is improved. If the cost value is improved, the proposal is accepted with a probability that depends on the random number *u*. The probability of acceptance depending on the fitness improvement is tabulated in Table 3. The reader can observe that the new candidate is almost always accepted when there is a significant improvement.

**Table 3:** Probability of acceptance depending on the fitness value improvement.

Fitness Improvement ($r_{log}$)	0.10	0.35	0.69	1.20	1.60	2.39	6.90
*p* (acceptance) (%)	10	30	50	70	80	90	99.99

Comparing this acceptance criterion to the previous one, the original DE-MC optimizer always accepts the new candidate if the probability is improved. In this filter, the new candidate is accepted if there is a significant improvement, and small improvements that can be caused by the noise are filtered, which reduces the optimization in the noise band. Besides, Ter Braak’s method can accept a proposal that does not improve the probability. In this algorithm, the proposal cannot be accepted if the fitness value is not improved. Despite this mechanism having been empirically fixed, these capabilities are more suitable for a GL filter according to our experience in this topic.

In each iteration, the previous steps are repeated for all candidates to create the whole population for the next iteration. Each population member can be viewed as a Markov chain that evolves to the locations with the best fitness values. The evolutionary loop continues until the convergence conditions are met (Line 19), returning the best member (Line 18), which is the solution of the GL problem.

The current method is not limited to a static robot. It also works with multiple motion-perception cycles (as most localization filters do). The process of integrating motion and sensor information is explained in [2]. The same method is applied here. The best solution is held as the robot location after convergence. If the robot moves to another place, the population set is moved according to the motion model $x_{t+1}=f\left(x_{t},u_{t}\right)$, where *t* represents the time instant when the robot receives information from its sensors (odometry information $u_{t}$ and laser readings $z_{t}$). The problem is now converted into the tracking one. The algorithm is executed when the robot is in the new location, but the initial population is formed by the results of the last execution of the algorithm.

## 6. Experimental Results

**Figure 4:**
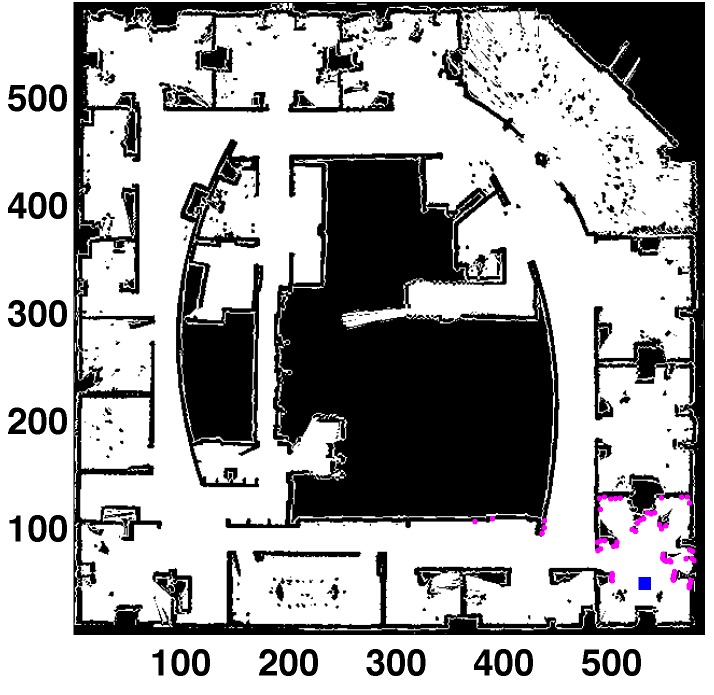
Global localization (GL) in a real map (Intel lab). All units in cells. Laser readings in purple. Robot’s location (531, 48, 85) in blue.

The localization filter has been tested in the real map shown in Figure 4 (thanks to Dieter Fox for making this dataset available). The area of this map is 29×29 m${}^{2}$, and the cell size is 5 cm. This is a medium-sized map with highly cluttered areas. The experiments use simulated scans in the real map (to obtain the laser readings from the true location). The uncertainty is added to these measurements to perform more realistic tests. The sensor noise has been modeled as a Gaussian distribution over the laser distance where the standard deviation specifies the noise. The laser scan is formed by 61 measurements separated by three degrees (180-degree field of view). The robot’s pose is defined by three parameters: the first coordinate corresponds to the horizontal axis; the second one is the vertical axis; and the third one represents the orientation (zero being pointing right, horizontal direction, increasing clockwise).

Three different cases have been analyzed: GL without motion (Section 6.1), GL and tracking along a path (Section 6.2) and GL in environments with occlusions (Section 6.3).

### 6.1. Global Localization

The algorithm performance when the robot is located in an unknown place of the environment is studied in the first experiment. The objective is to examine the capabilities of the filter in a single motion-perception cycle, which means that the robot’s pose is estimated using a single laser scan. This condition can be viewed as a particular case of the GL problem where it will not be possible to distinguish between two places with the same (laser) appearance, a situation that often happens in indoor structured environments. This situation is equivalent to the well-known kidnapping problem when this filter is applied, because the population is generated randomly if there is no initial information about the robot’s location.

The results when the robot is located in random places of the real map of Figure 4 are registered in Table 4. $e_{d}$ is the position error, and $e_{\theta }$ is the orientation error.

**Table 4:** Error and success rate for different random places. Sensor noise: standard deviation of 1%. $S_{r}$ in %. Errors as the mean ± standard deviation.

Location	$N_{\mathrm{occ}}$	$S_{r}$ (%)	$e_{d}$(mm)	$e_{\theta }$ (${}^{\circ }$)
(320, 460, 10)	100	100	15.6342±16.6850	0.1224±0.1235
(531, 48, 85)	180	68	9.5033±6.4544	0.1531±0.1215
(440, 322, 21)	60	100	17.9249±13.8110	0.0882±0.0626
(75, 60, 3)	240	100	30.6244±19.5952	0.3046±0.1575
(210, 401, 167)	140	74	9.2479±4.3952	0.1042±0.0623
(351, 477, 99)	100	100	10.2392±8.2712	0.1533±0.1078

The “success rate” ($S_{r}$) has been defined to study the robustness of the method (viewed as the chances of success). There are two options when estimating the robot’s pose from a specific location: the estimate coincides with the true pose (success) or they are different (failure). $S_{r}$ is measured after executing the GL module multiple times for the same location. The success rate is equal to the number of times in which the algorithm returns the correct pose divided by the total number of runs. It is possible to fix a distance threshold to distinguish between success and failure because the true pose is known. This distance is 50 cm in these experiments. $S_{r}$ is given in %. For example, $S_{r}=100$ when the algorithm is run 50 times, and the true pose is obtained in all cases.

The localization filter is highly dependent on the population size. $N_{P}$ has to be large enough to be successful in most cases, but higher values have a negative effect on the computational cost. For simplicity, only the errors with an optimum population size (lowest $N_{P}$ with maximum $S_{r}$) are given. A more detailed review about the influence of this parameter can be found in [11].

Several factors have to be considered when choosing the population size. The population requirements depend on the size of the sensing area perceived in a laser scan. Smaller populations will be needed for larger areas. This fact is related to the basin of attraction of the local minimum, which is larger for larger areas. The map size is another variable that has to be taken into account. There are other factors that have to be analyzed, such as the number of symmetries, the sensor information and the occlusions. An exhaustive study about the population requirements is given in [47].

Regarding the success rate, $S_{r}=100$ in all cases without perceptual ambiguities. There are two cases where $S_{r}$ is not maximum because the mobile robot is inside an office and there are several places with a similar appearance. They were chosen because the localization process is harder in these locations. These spots correspond to the offices located on the right side of the figure (531,48,85) and the rooms in the upper left corner (210,401,167). The similarities between places can be appreciated in the map. More readings from different locations are needed to solve the ambiguities. The population sizes are similar to those obtained in [11], which are lower than the requirements of the DE-based original filter.

The localization error is in the interval (9.24 mm, 30.62 mm) in position and ($0.08^{\circ },0.30^{\circ }$) in orientation. These errors are similar to those reported in our previous work, and they are low enough to conclude that the GL task is efficiently accomplished. Reviewing the state of the art, we have not found other methods with lower errors. For example, the position errors are in the range of cm in [15,17,23,30]. However, it is not easy to make a fair comparison, because many different parameters that are not considered, such as the sensor resolution or the map size, could be involved.

### 6.2. Global Localization and Pose Tracking

The algorithm performance when the robot is moving is tested in this section. This is a typical condition in robotic applications, because the robot is usually moving and receiving motion and perceptive information from multiple locations. The case reviewed in Section 6.1 corresponds to a more critical situation regarding the amount of available information. If there are two places with the same appearance that cannot be distinguished from one another, the algorithm must keep both hypotheses, and additional measurements are needed to estimate the correct location.

In this procedure, the population is decreased to 20 after convergence, and the maximum number of iterations is also limited. The purpose of pose tracking is a fast response, because it is assumed that the robot was correctly localized at the beginning of the path. These parameters could easily be changed if the requirements include obtaining the maximum accuracy.

**Figure 5:**
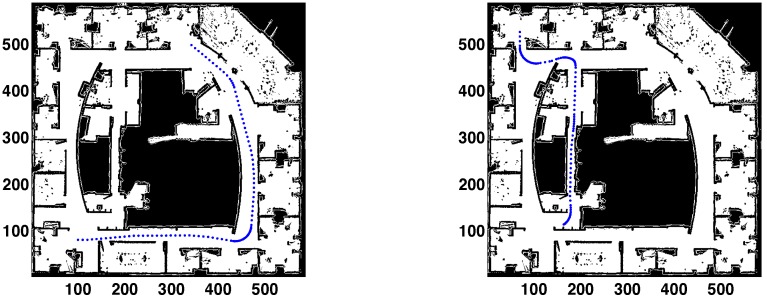
(**Left**) Path 1. Starting point: bottom left corner; (**Right**) Path 2. Starting point: upper left corner.

**Figure 6:**
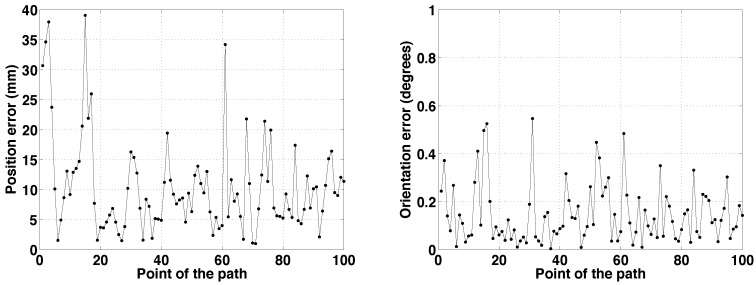
Path 1. Errors *vs*. point of the path. (**Left**) Position error; (**Right**) orientation error.

**Figure 7:**
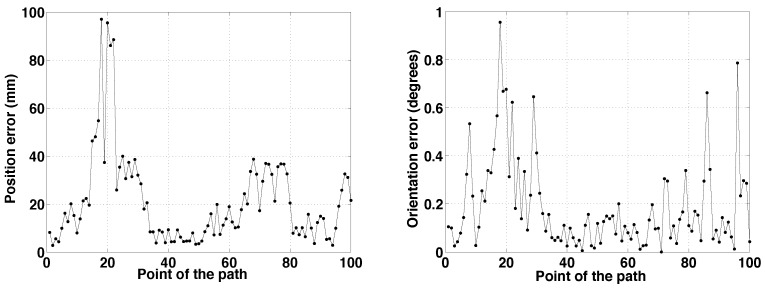
Path 2. Errors *vs*. point of the path. (**Left**) Position error; (**Right**) orientation error.

The results for two different paths are presented in Figure 5, Figure 6 and Figure 7. Analyzing Figure 5, each path is formed by 100 points. Path 1 starts in the bottom left corner, with coordinates (100,80,0). The robot is traversing the main corridor during the whole path. First, moving from left to right; after that, turning left and crossing the right side corridor. The final pose is (343,497,140). In Path 2, the robot starts from the upper left corner, which is an office with coordinates (70,525,270). It exits to the hallway and then makes an S-turn to visit the narrow corridor in the middle of the map. The path terminates at the lower end of the corridor, in (164,113,200).

The robot is correctly localized after the first motion-perception cycle, which is a clear advantage with respect to other classic localization approaches, such as those based on MC sampling techniques. Observing the evolution of the errors in Figure 6 and Figure 7, the initial position errors are 30.7 mm and 8.2 mm, respectively.

When Path 1 is examined, the position error is lower than 40 mm during the whole path. The larger errors could be caused by sharp turns, larger sensing areas or higher noise in the sensor measurements. The localization process is harder when the robot is turning, because there are more significant changes in the perceptive information. Nevertheless, as said before, the convergence conditions could be changed to obtain more accurate results when necessary.

For Path 2, the upper limit of the position error is close to 100 mm in some points. These points correspond to the S-turn. After that, the upper limit of the error decreases to 40 mm.

The orientation error is lower than one degree during the whole path in both cases, which is low enough to conclude that the localization process is solved in an adequate way.

When these results are compared to those obtained by other research groups, Zhang *et al.* [17] have contrasted their SAMCL method with the basic MC localization technique. They have conducted experiments measuring the localization error in a quasi-symmetrical corridor of a simulated indoor environment. Their method outperforms the classic version of MC in pose tracking, GL and robot kidnapping. Regarding the localization error, the main difference between their method and the technique presented here is that, for the SAMCL algorithm, worse results are obtained at the beginning of the path. Their initial errors are around 80 cm. When the robot is properly localized, their errors are lower than 20 cm. However, as previously said, a fair comparison between tracking errors is not possible, because there are other aspects that have to be considered.

The computational time depends basically on the number of iterations (iter), the population size ($N_{P}$) and the number of measurements of the laser scan (61 in these experiments). The number of iterations is higher, and the population size is larger in GL than in tracking. If the computational times of this experiment are analyzed, the required time for GL (first motion-perception cycle) is 37.02 s for Path 1 (iter=1125 and $N_{P}=144$). In tracking, the average time for the same path is 0.73 s (average iterations iter=41.11 and $N_{P}=20$). For Path 2, GL requires 61.18 s (iter=1526 and $N_{P}=194$). In tracking, the average time is 1.13 s (average iterations iter=150.32 and $N_{P}=20$). Depending on the velocity of the robot and the time between laser scans, the algorithm could be used in real-time applications when the robot is correctly localized (tracking). The current version is implemented in MATLAB in a computer with a 2.7 GHz Intel Core i7 processor.

### 6.3. Occlusions

Two different situations are proposed to examine the method performance in environments with occlusions. First, the laser measurements will be contaminated by uniform noise. Second, unmodeled obstacles will be included in the real observations.

#### 6.3.1. Uniform Noise

A great feature of any GL module is the ability to obtain the robot’s location when there are mobile objects, people or unexpected obstacles that are not included in the real map. These unmodeled obstacles introduce contaminated measurements (and occlusions) in the information vector obtained by the sensing system that can be viewed as uniform noise. This situation was previously analyzed in [5] using the original DE-based filter. The same experiment has been conducted here to compare the KL-based cost function to the L2-norm.

The laser scan has been contaminated with a uniform distribution located between 25% and 75% of the distance measured. The next equation is applied to introduce the contamination noise in each laser reading:

(18) $$z_{k,c}=\left(1-\epsilon \right)N\left(z_{k},\sigma \right)+\epsilon U\left(0.25z_{k},0.75z_{k}\right)$$

where $z_{k,c}$ is the contaminated measurement, *ϵ* is the contamination level, $N(z_{k},\sigma )$ is the sensor noise probability distribution centered at the true measurement and $U(0.25z_{k},0.75z_{k})$ is a uniform probability distribution in the interval $\left(0.25z_{k},0.75z_{k}\right)$. The contamination level is a random number equal to zero or one depending on the percentage of measurements that will be contaminated. In other words, the laser measurement will be given by *N* (not contaminated) or *U* (contaminated), depending on the value of *ϵ*.

The algorithm has been tested when the robot’s location is (320,460,10) and the sensor noise *σ* is 1%. A single observation vector is used because motion is not considered.

**Figure 8:**
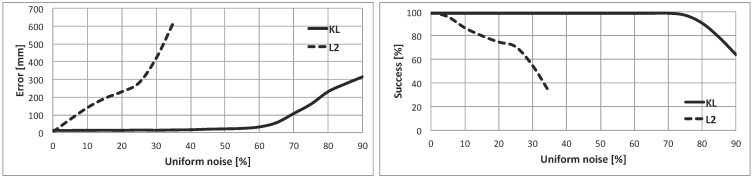
(**Left**) GL error *vs*. uniform noise (percentage of contaminated measurements); (**Right**) success *vs*. uniform noise.

The results of this experiment are registered in Figure 8. In the left part, the localization error is plotted against the contaminated noise (contaminated measurements divided by the total number of measurements, in percentage). In the right part, the percentage of success ($S_{r}$) is represented against the contaminated noise.

**Figure 9:**
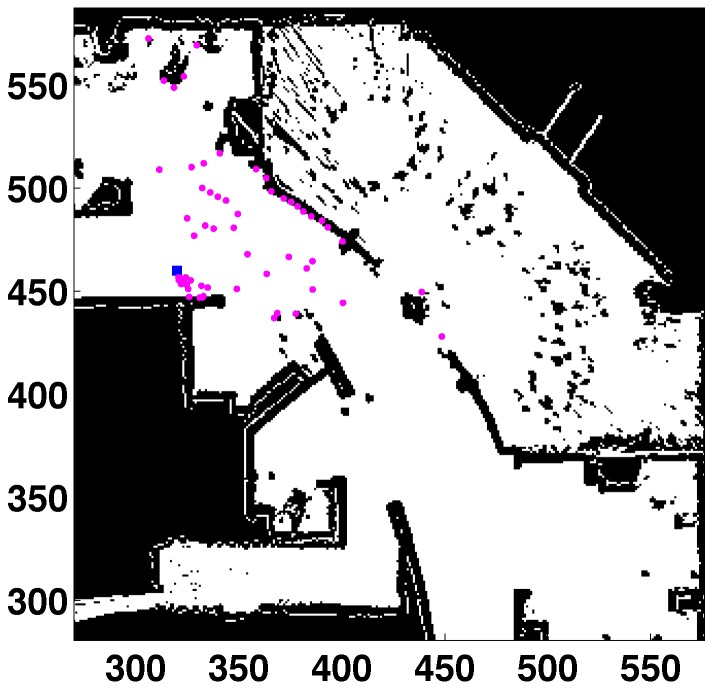
Example of contaminated laser scan with 55% contamination. All units in cells. Laser readings in purple. Robot’s location (320,460,10) in the blue square.

If the shape of the error curve is studied, the KL-based method clearly outperforms the quadratic-based one. The error is almost constant up to 55% of contamination, which is a great result, because it means that 55 out of every 100 readings are wrong measurements originated by the contamination (Figure 9). The error of the KL-based option when the contaminated noise is 55% is lower than 25 mm, which is a very low value. For the L2-norm, the error is significantly worse even with low levels of contamination. Besides, the method fails when the percentage of contamination is greater or equal to 35%.

Similar conclusions can be drawn from the percentage of the success plot. For the KL-based technique, the method success is maximum (100%) even with a high level of contamination (up to 75%). For the quadratic-based method, $S_{r}$ decreases significantly when the contamination level is increased. For example, the percentage of success is 71% when the uniform noise is 20%.

When the current results are compared to those presented in [5], it is possible to conclude that the great performance of the KL-based cost function has been inherited by this filter. In fact, the results when the KL divergence is implemented in the current method are even better than the previous features of the basic version of the DE algorithm. Both indicators, the position error and the percentage of success, are less affected by the contamination level in the experiments shown in this paper. The GL filter will be adequate for dynamic environments with plenty of people and moving objects that are not registered in the known map.

#### 6.3.2. Unmodeled Obstacles

The purpose of this section is to analyze the performance of the method when unmodeled obstacles of a fixed size are included in the scanned area. Two different experiments have been developed. The objective is again to compare the KL-based method to the quadratic-based one.

**Figure 10:**
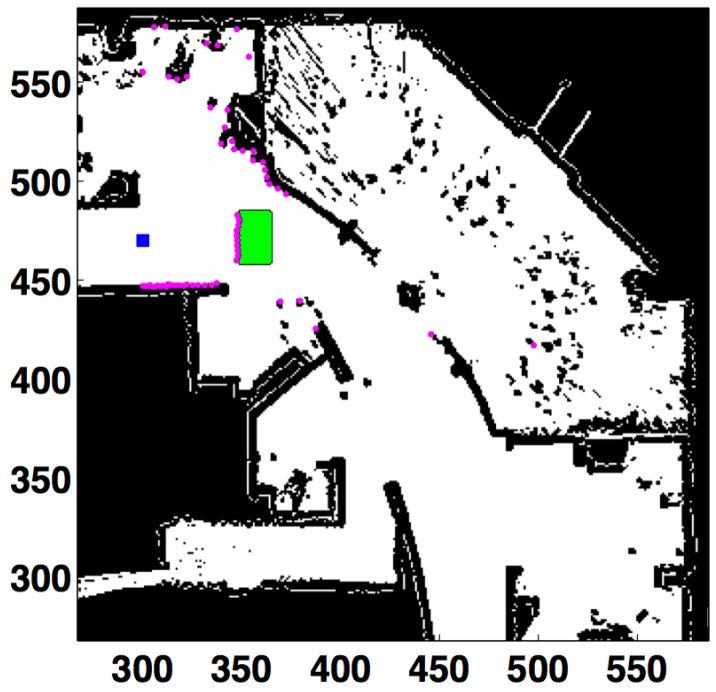
Obstacle produces an occlusion in front of the robot. All units in cells. Laser readings in purple. Robot’s location in the blue square. Unmodeled obstacle in green.

The first test consists of including a big unmodeled obstacle centered around coordinates (357,470). The percentage of success is measured depending on the distance to the obstacle. The localization process is harder, because a big area of the map cannot be seen when the robot is close to the obstacle. This situation is illustrated in Figure 10, which shows the laser measurements when the robot is 48 cells (2.4 m) away from the obstacle. It can be observed that the scanned area has been drastically changed by the unmodeled obstacle.

Table 5 (KL) and Table 6 (L2) detail the results when the robot is approaching the obstacle. $d_{obs}$ is the distance to the obstacle, and $N_{occ}$ represents the number of measurements originated by the unmodeled obstacle. Comparing both tables, the minimum distance $d_{obs}$ with optimum results ($S_{r}=100\%$) is 148 cells (7.4 m) for the L2-based filter and 48 cells (2.4 m) for the KL-based one. Besides, the method presented in this paper has succeeded in many cases ($S_{r}=80\%$) even when the robot is very close to the obstacle (eight cells, 0.4 m, and 39 erroneous readings). The L2-based technique fails with only four wrong measurements.

**Table 5:** KL-based differential evolution (DE)-MC GL when approaching an unmodeled obstacle. Errors are the mean ± standard deviation. Sensor noise: 1%.

Location	$d_{\mathrm{obs}}$(cells)	$N_{P}$	$N_{\mathrm{occ}}$	$S_{r}$ (%)	$e_{d}$(mm)	$e_{\theta }$ (${}^{\circ }$)
(300,470,0)	48	178	10	100	4.3613±3.0364	0.0992±0.0859
(310,470,0)	38	206	13	96	16.4604±13.3210	0.2204±0.1779
(320,470,0)	28	239	17	96	16.5246±11.0114	0.1773±0.1169
(330,470,0)	18	299	25	96	10.6058±8.2331	0.1218±0.1045
(335,470,0)	13	327	31	92	5.6528±4.9463	0.1115±0.1083
(340,470,0)	8	413	39	80	11.6118±8.0695	0.1354±0.0753

**Table 6:** L2-based DE-MC GL when approaching an unmodeled obstacle. Errors are the mean ± standard deviation. Sensor noise: 1%.

Location	$d_{\mathrm{obs}}$(cells)	$N_{P}$	$N_{\mathrm{occ}}$	$S_{r}$ (%)	$e_{d}$(mm)	$e_{\theta }$ (${}^{\circ }$)
(190,470,0)	158	159	3	100	16.6705±7.3874	0.2968±0.0607
(200,470,0)	148	217	3	100	5.3944±2.998	0.2376±0.0361
(202,470,0)	146	213	4	28	60.3100±43.6562	0.5479±0.0594
(203,470,0)	145	213	4	0	-	-

**Figure 11:**
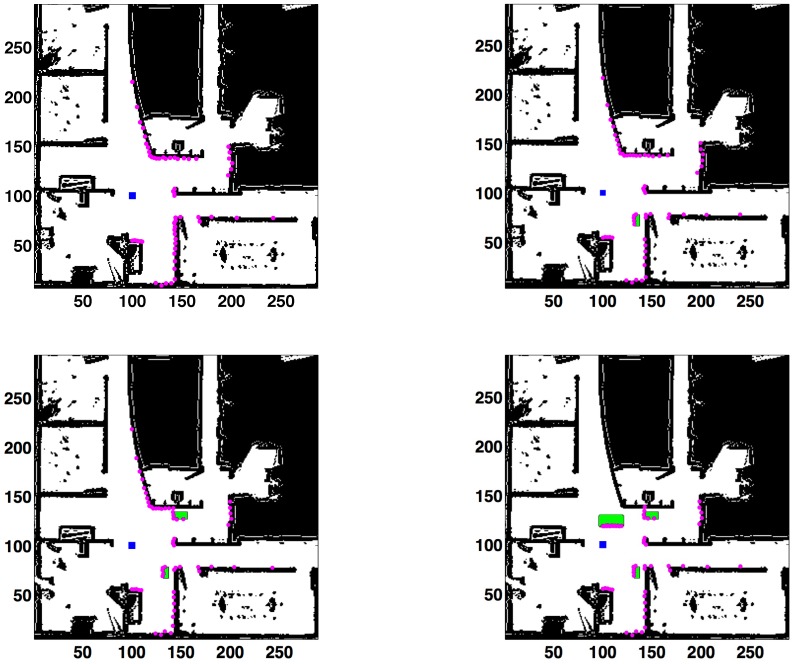
Occlusions originated by small unmodeled obstacles. Laser readings in purple. Robot’s location (100,100,0) in the blue square. Number of unmodeled obstacles, from left to right and top to bottom: zero (**left (top)**), one ($N_{occ}=5$) (**right (top)**), two (10) (**left (bottom)**), three (26) (**right (bottom)**). Unmodeled obstacles in green.

The next experiment consists of adding small unmodeled obstacles to the scanned area. The robot is in (100,100,0) and different objects that are not included in the known map are introduced in the sensing area. Four different cases are studied, corresponding to zero, one, two or three unmodeled obstacles (Figure 11). The erroneous readings are 0, 5, 10 and 26, respectively. The results are detailed in Table 7 (KL) and Table 8 (L2).

**Table 7:** KL-based DE-MC GL with different unmodeled obstacles. Robot’s location: (100, 100, 0). Errors are the mean ± standard deviation. Sensor noise: 1%.

$N_{\mathrm{occ}}$	$S_{r}$ (%)	$e_{d}$(mm)	$e_{\theta }$ (${}^{\circ }$)
0	100	20.9562±16.4348	0.1168±0.0883
5	100	19.3654±10.1017	0.0819±0.0920
10	100	13.6289±9.0097	0.0790±0.0720
26	100	7.0735±4.4406	0.1538±0.0942

**Table 8:** L2-based DE-MC GL with different unmodeled obstacles. Robot’s location: (100, 100, 0). Errors are the mean ± standard deviation. Sensor noise: 1%.

$N_{\mathrm{occ}}$	$S_{r}$(%)	$e_{d}$(mm)	$e_{\theta }$ (${}^{\circ }$)
0	100	5.0131±3.4633	0.0498±0.0380
5	100	36.6119±11.4466	0.3688±0.1241
10	100	53.6751±6.0826	0.1103±0.0744
26	96	266.7144±21.9948	4.3377±0.3632

When both tables are compared, the errors in all cases with unmodeled obstacles are lower when the KL-based method is applied. In addition, the L2-based filter does not find an accurate solution when the number of occlusions is 26. Therefore, it can be concluded that the KL-based approach is a more suitable option for this type of environment.

If Table 7 is analyzed, the position error is in the interval (7.07 mm, 20.96 mm). The unmodeled obstacles do not worsen the error. In fact, the lowest position error is obtained with three obstacles (but the difference is not significant; the results are given in mm). For the L2-norm in Table 8, the error is much lower without unmodeled obstacles.

Finally, it should be emphasized that the performance of the method is excellent when unmodeled obstacles are added to the sensing area.

## 7. Conclusions

The GL problem for a mobile robot is addressed in this paper by applying a technique that relies on a combination of the DE evolutionary method and the population-based MCMC algorithm. As demonstrated in [11], the DE-MC GL module keeps the statistical robustness of the MCMC technique and the exploration properties of the evolutionary filter.

The localization filter minimizes a cost function that uses the information provided by the sensing system of the mobile robot to estimate the robot’s location in a known map. Most localization methods are based on symmetric cost functions. In this work, the cost function considers the KL divergence, which allows one to treat the sensor information asymmetrically. This change in the fitness function causes a great improvement in environments with occlusions.

Different experiments have been carried out in a real map to test the algorithm. The localization error and the success rate have been measured in different situations.

First, the robot has been located in different places, and the performance is studied in a single motion-perception cycle (with a single laser scan). The GL method obtains accurate solutions in all cases, and there are no disadvantages when compared to previous versions of the filter. The number of particles needed to obtain optimum results and the success rates are similar to those presented in [11]. Therefore, important characteristics are inherited from the previous version of the DE-MC GL module. The chances of success (strongly related to the parameter defined as the success rate) are improved with respect to the DE-based filter, making this method a more suitable approach in challenging environments. The population requirements are much lower when compared to the DE-based filter.

The performance of the method has also been analyzed when the robot is moving (multiple motion-perception cycles). The main advantage with respect to classic MC-based approaches is that the correct location can be obtained even after a single motion-perception cycle. After that, the localization error is maintained at a low value during the whole path.

Finally, the algorithm behavior in environments with occlusions has been studied in different tests. The goal is the same followed in [5], which is to check if the asymmetric processing of the sensor information (KL divergence) enhances the capabilities of the basic option (L2-norm).

In the presence of contaminated noise, the KL-based method clearly outperforms the L2-based one. For the KL-based technique, the success rate is maximum, even with high levels of contamination. These excellent results have important implications. The current method will be a promising approach for dynamic environments with people and moving obstacles, which is a very typical situation.

Similar conclusions can be reached when unmodeled obstacles are added to the sensing area. If a big obstacle is placed in front of the robot, the GL module succeeds even when the robot is very close to the obstacle. If small obstacles are added to the scanned area, the errors in all cases with unmodeled obstacles are lower when the new technique is applied. It can be concluded that the KL-based DE-MC filter is an outstanding method for environments with occluded areas.

Some aspects to be addressed in the future are a detailed study about the convergence properties, with a strong influence on the computational cost, and experiments in real-time in zones with dynamic obstacles.
